# Supplementary material for: Changes in Resurgent Sodium Current Contribute to the Hyperexcitability of Muscles in Patients with Paramyotonia Congenita
Source: Biomedicines. 2021 Jan 8;9(1):51. doi: 10.3390/biomedicines9010051 (PMC7826575; doi:10.3390/biomedicines9010051)
Supplement: Supplementary file 1 [file biomedicines-09-00051-s001.pdf]

Supplementary materials

Article

Changes in resurgent sodium current contribute to the hyperexcitability of muscles in patients with paramyotonia congenita

Chiung-Wei Huang,[1,2] Hsing-Jung Lai,[3,4] Pi-Chen Lin,[5] Ming-Jen Lee [3, 6 and *]

1. Department of Post Baccalaureate Medicine, Kaohsiung Medical University, Kaohsiung, Taiwan. g10054b@ms51.hinet.net
2. Department of Physiology, Kaohsiung Medical University, Kaohsiung, Taiwan. g10054b@ms51.hinet.net
3. Department of Neurology, National Taiwan University Hospital, Taipei, Taiwan. i5492111@gmail.com and mjlee@ntu.edu.tw
4. Department of Neurology, National Taiwan University Hospital Jinshan Branch, New Taipei City, Taiwan. i5492111@gmail.com
5. Division of Endocrinology and Metabolism, Department of Internal Medicine, Kaohsiung Medical University Hospital, Kaohsiung, Taiwan. pichli@kmu.edu.tw
6. Department of Neurology, National Taiwan University Hospital Yunlin Branch, Yunlin, Taiwan. mjlee@ntu.edu.tw

* Correspondence: mjlee@ntu.edu.tw; Tel.: +88-62-2312-3456 (ext 65336)

Figure S1

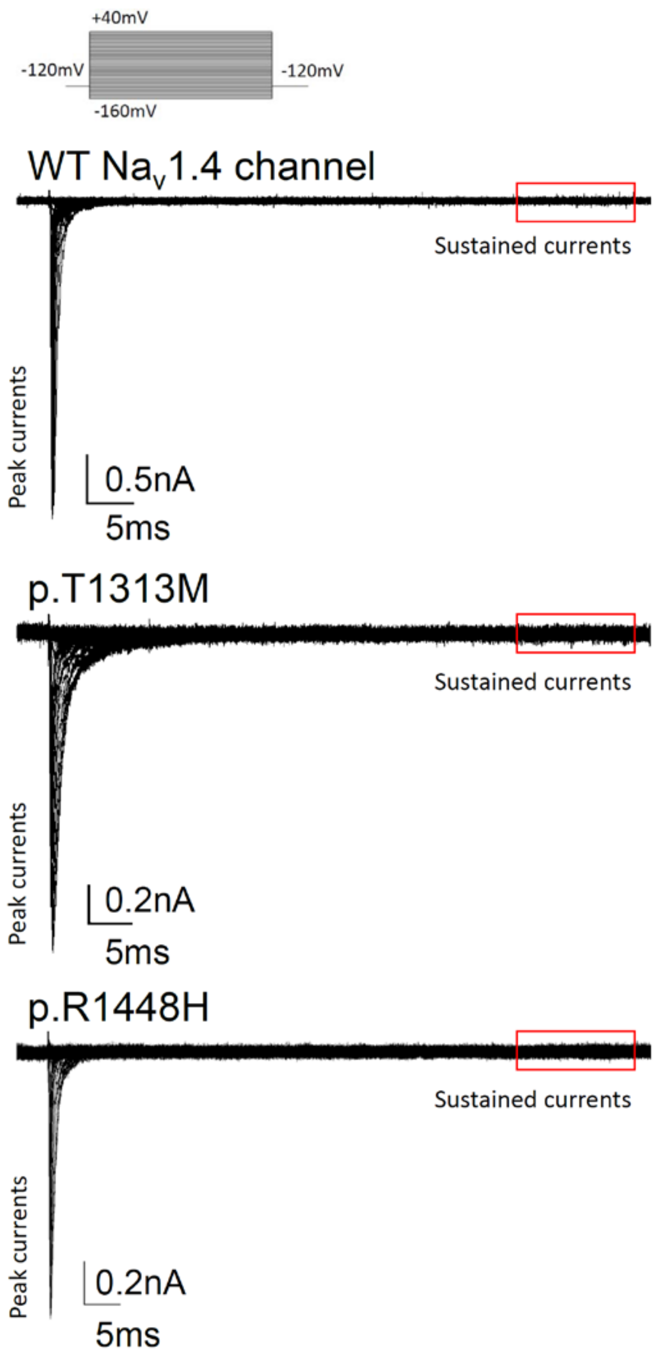

Figure S1. Sample sweeps shows the peak currents and sustained currents in WT, p.T1313M ,and p.R1448H mutant Na_v1.4 channels
